# Evaluation of an AAV2-Based Rapamycin-Regulated Glial Cell Line-Derived Neurotrophic Factor (GDNF) Expression Vector System

**DOI:** 10.1371/journal.pone.0027728

**Published:** 2011-11-21

**Authors:** Piotr Hadaczek, Janine Beyer, Adrian Kells, Wade Narrow, William Bowers, Howard J. Federoff, John Forsayeth, Krystof S. Bankiewicz

**Affiliations:** 1 Department of Neurosurgery, University of California San Francisco, San Francisco, California, United States of America; 2 Department of Neurology, Center for Neural Development and Disease, University of Rochester Medical Center, Rochester, New York, United States of America; 3 Departments of Neurology and Neuroscience, Georgetown University Medical Center, Washington, D.C., United States of America; University of South Florida, United States of America

## Abstract

Effective regulation of transgene product in anatomically circumscribed brain tissue is dependent on the pharmacokinetics of the regulating agent, the kinetics of transcriptional activation and degradation of the transgene product. We evaluated rapamycin-regulated AAV2-GDNF expression in the rat brain (striatum). Regulated (a dual-component system: AAV2-FBZhGDNF + AAV2-TF1Nc) and constitutive (CMV-driven) expression vectors were compared. Constitutively active AAV2-GDNF directed stable GDNF expression in a dose-dependent manner and it increased for the first month, thereafter reaching a plateau that was maintained over a further 3 months. For the AAV2-regGDNF, rapamycin was administered in a 3-days on/4-days off cycle. Intraperitoneal, oral, and direct brain delivery (CED) of rapamycin were evaluated. Two cycles of rapamycin at an intraperitoneal dose of 10 mg/kg gave the highest GDNF level (2.75±0.01****ng/mg protein). Six cycles at 3 mg/kg resulted in lower GDNF values (1.36±0.3 ng/mg protein). Interestingly, CED of rapamycin into the brain at a very low dose (50 ng) induced GDNF levels comparable to a 6-week intraperitoneal rapamycin cycle. This study demonstrates the effectiveness of rapamycin regulation in the CNS. However, the kinetics of the transgene in brain tissue, the regulator dosing amount and schedule are critical parameters that influence the kinetics of accumulation and zenith of the encoded transgene product.

## Introduction

One promising disease-modifying strategy for Parkinson's Disease (PD) is the provision of neurotrophic factors for protection and restoration of the damaged dopaminergic (DA) innervation of the basal ganglia [1], [2], [3], [4], [5], [6], [7], [8]. The most clinically advanced family of factors is defined by the prototype, glial cell line-derived neurotrophic factor (GDNF). Based upon strong preclinical data of its protective effects on DA neurons, several clinical trials were undertaken in which recombinant GDNF was infused either into cerebral ventricles [9], [10] or into the putamen [11], [12] through an indwelling catheter. The significance of these clinical studies remains controversial [12], [13], [14], [15], [16], although apparent efficacy was reported in one trial [11], [12]. One issue identified in these studies was whether the infused GDNF was adequately localized to the target region. The fact that some trial participants developed anti-GDNF antibodies [11], [12] and that a few treated non-human primates (NHP) showed cerebellar pathology [17], suggests that GDNF protein delivery was sub-optimal. Nevertheless, parenchymal GDNF infusion has the significant advantage that treatment can be terminated if necessary, a feature unavailable to clinical gene transfer at present. GDNF gene therapy, however, is attractive because it provides an effectively localized expression of GDNF at levels many orders of magnitude lower than protein infusions with impressive efficacy in Parkinsonian nonhuman primates (NHP) in both neuroprotective [18] and neurorestorative [8], [19] paradigms. The possibility, therefore, of developing a regulated gene therapy vector is highly attractive since it promises to combine localized GDNF delivery with the capacity to adjust steady state levels through exogenous administration of a brain-penetrant small molecule regulator.

Regulated gene expression can be achieved via a chimeric gene construction linking a regulatory *cis* element upstream of the gene to be transcribed. One such strategy is to use a drug that can cross the blood-brain barrier to act on drug-dependent promoters that directly activate or repress target gene transcription. The rapamycin-regulated transcriptional control system uses rapamycin (or analogs) to dimerize the activation (p65) and DNA-binding (ZFHD1) domains of a chimeric transcription factor through the rapamycin-binding domains of the proteins FRAP and FKBP. The activated transcription factor then binds to zinc finger motifs upstream of a minimal promoter (from human interleukin-2) and drives expression of the encoded transgene [20]. In the case of GDNF, regulated expression is kinetically complex because the protein has a prolonged tissue clearance [21]. There is, therefore, no simple relationship between activation of transcription, protein biogenesis, secretion and subsequent extracellular accumulation of GDNF in the transduced brain region (striatum).

As part of an effort to effectively translate a regulated gene transfer platform into clinical studies we undertook the studies reported below. These experiments address the kinetics of GDNF biogenesis and accumulation with various rapamycin dosing regimens with a rapamycin-regulated AAV2-GDNF vector system (AAV2-regGDNF) delivered into the rat striatum. It should be noted that this study examined only the kinetics of this system and not its therapeutic implications. We found that 2 cycles of 3 days on/4 days off rapamycin at an intraperitoneal dose of 10 mg/kg, yielded a high GDNF level (2.75±0.01****ng/mg protein). In turn, six cycles of lower dose, 3 mg/kg rapamycin [22], resulted in lower GDNF levels (1.36±0.3 ng/mg protein). Further studies in Parkinsonian NHP should reveal whether such dosing is well tolerated and able to promote dopaminergic regeneration.

## Materials and Methods

### Construction and preparation of the constitutive AAV2-GDNF

This vector was previously described in studies by Johnston et al. [23]. Three different concentrations were chosen for the time-course study: 1.1×10^13^ vg/ml; 6.05×10^12^ vg/ml; and 1.1×10^12^ vg/ml. The control vector, AAV2-LacZ, was described previously [24]

### Construction and preparation of the rapamycin-regulated AAV2-GDNF

The constitutively expressed repressor/transactivator plasmid, pFBGR(TF1Nc.3), was constructed by excising the CMV.TF1Nc.3 expression cassette from pAAV.CMV.TF1Nc.3 (Avigen, Alameda, CA) with NotI. This cassette was inserted into NotI-digested pFBGR [25]. The rapamycin-regulated plasmid pFBZ(hGDNF) was constructed by first creating a shuttle plasmid harboring the Z12I promoter, termed pBSZ12I. The Z12I promoter was removed from pAV.Z12I.AADC (Avigen, Alameda, CA) using NotI and ClaI and then exchanged with the CMV promoter of the shuttle plasmid pBSFBRmcs [26] to create pBSZ12I. Creation of pBSZ12I(hGDNF) was done by removal of the hGDNF coding sequence from pAV.Z12I.hGDNF (Avigen, Alameda, CA) with ClaI and ligation into ClaI-digested pBSZ12I. The final pFBZ(hGDNF) plasmid was generated by excision of the expression cassette from pBSZ12I(hGDNF) with NotI and insertion into NotI-digested pFBGR.

Recombinant AAV (2 L total culture volume per vector) was generated via a baculovirus-based method described previously [25]. Cultures were processed through 3 freeze/thaw cycles consisting of an overnight freeze at –80°C and a 2-h thaw at 37°C prior to purification and concentration. After cell lysis, cultures were centrifuged at 1290 x g for 10 min to remove cellular debris. Crude supernatants were subsequently treated with Benzonase (20 units/ml; Sigma-Aldrich, St. Louis, USA) and incubated at 37°C for 1 h. Treated crude supernatants were filtered through 0.2-µm polyethersulfone membranes prior to purification.

Viruses were purified on a Biologic DuoFlow FPLC (BioRad, Hercules, CA) with an attached XK50/20 column containing 70 ml of AVB Sepharose chromatography media (GE Healthcare, Piscataway, NJ), as previously described [27] with the following modifications. Flow rates were established at 2.7 ml/min. Elution fractions were collected in sterile tubes containing 0.5 ml of sterilized Tris, pH 8.0, to neutralize citrate acidity. Peak virus-containing fractions, determined by OD_280_ measurement, were pooled and concentrated to 2 ml with VivaSpin 20 ultraconcentration cassettes (MWCO 100,000; Sartorius-Stedim, Bohemia, NY). Concentrated virus was dialyzed for 2 h in 2 L of sterilized 1x PBS with 2 mM MgCl_2_, dispensed and frozen at −80°C. Viral titers were determined by quantitative real-time PCR [8] with primer/probe sets specific for the Z12I or CMV promoter sequence. Rapamycin-induced expression of hGDNF was confirmed by transduction of HEK293A cells (Invitrogen, Carlsbad, CA) and ELISA-based measurement of hGDNF protein concentrations (Promega, Madison, WI). The titers of both vectors were 5.5×10^12^ vg/ml. They were mixed 1∶1 before infusion into rat brain.

### Animals

Experiments were performed with male Sprague-Dawley rats (Charles River Laboratories; Wilmington, MA) each weighing 350–400 g. All procedures were approved by, and in accordance with, the regulations of the Institutional Animal Care and Use Committee of the University of California, San Francisco (approval number: AN082508), and all efforts were made to minimize suffering.

### Stereotactic surgery

**Figure 1:**
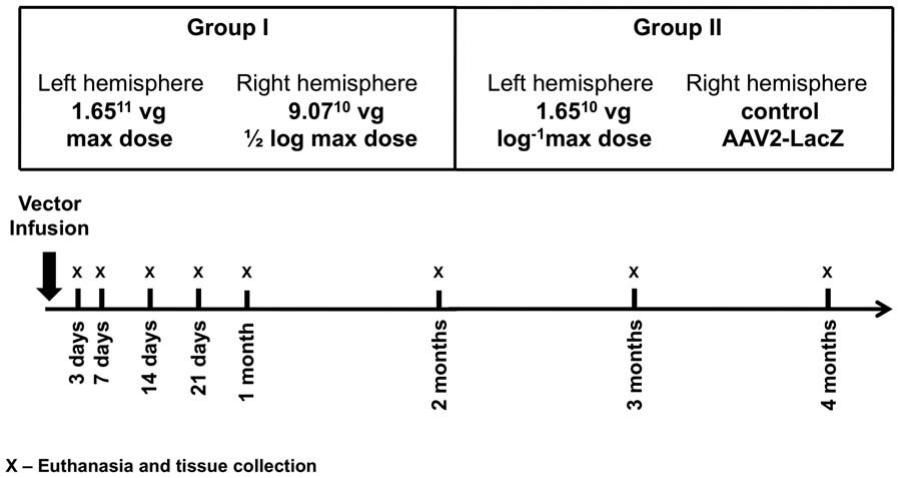
Study design for time-course of GDNF expression in the rat brain after transduction with the constitutive AAV2-GDNF.

We infused both vector systems, constitutive AAV2-GDNF and rapamycin-regulated (AAV2-FBZhGDNF and AAV2-TF1Nc at a 1∶1 ratio) into the rat striatum bilaterally (total volume 15 µl for 30 min; the infusion rate was 0.5 µl/min) by convection-enhanced delivery (CED) as previously described [21]. For the time-course study with the constitutive AAV2-GDNF, 84 rats divided into two groups were used. Rats from Group I (n = 42) were infused with the maximum dose of the vector, 1.65×10^11^ vg, into their right hemispheres, while the left hemispheres were injected with 1/2 log of the maximum dose, 9.07×10^10^ vg. In turn, rats from Group II (n = 42) were injected with 10-fold less than the maximum dose, 1.65×10^10^ vg into the left hemisphere and 1.65×10^11^ vg of the control vector AAV2-LacZ into the right hemisphere. At each time point (3 days, 7 days, 14 days, 21 days, 1 month, 2 months, 3 months, and 4 months post-transduction) 5 rats from each group were euthanized and their brains were rapidly removed and the striatum was dissected bilaterally and immediately frozen for further processing (**Fig. 1**). To visualize the extent of GDNF expression, 2 rats from Group I and 2 rats from Group II were maintained alive for 4 months at which time their brains were processed for immunostaining for GDNF as described previously [21].

For the time-course study with rapamycin-regulated AAV2-GDNF (AAV2-regGDNF), 22 rats were infused (CED) bilaterally with 15 µl of a 1∶1 mixture of two vectors: AAV2-FB-hGDNF (2.12×10^12^ vg/ml) and AAV2-FB-ZTFINc3 (2.12×10^12^ vg/ml). Thirty days after transduction, rats received daily doses of either rapamycin (10 mg/kg) or vehicle (control) for 3 consecutive days. Animals were euthanized at 1, 4, 8, 14, 21, and 28 days after rapamycin administration. Six striata from 3 rats at each time point were extracted and processed for GDNF ELISA. Three control rats were given rapamycin excipient and euthanized on day 1. Similarly to the Group I for the constitutive AAV2-GDNF, brains of 2 rats euthanized 4 days after rapamycin treatment were processed for the immunohistochemical evaluation of GDNF expression [21].

For the testing of different dosing regimens of rapamycin, 33 rats were used (7 groups for intraperitoneal (i.p.) injections; 2 groups for oral administration; 1 group for direct brain infusion (CED); and 1 control group for vector infusion only without rapamycin dosing). Although only 3 animals per group were used, each animal provided 2 striata and the data, therefore, are the mean ± SEM of 6 determinations. In addition, 2 rats were tested for endogenous (baseline) levels of GDNF in the striatum (see schematic in **Table 1**).

**Table 1:** Summary of *in vivo* GDNF induction from the AAV2-regGDNF after various dosing regimens.

*Rapamycin regime*	*Striatal GDNF*ng/mg protein
Endogenous GDNF	0
**Intraperitoneal administration**	
**A**. Vector only	0.05±0.01
**B**. 1 × (3×10 mg/kg)	0.54±0.25
**C**. 2 × (3×10 mg/kg)	**2.75±0.01**
**D**. 1 × (3×1 mg/kg)	0.1±0.01
**E**. 3 × (3×1 mg/kg)	0.13±0.02
**F**. 1 × (3×3 mg/kg)	0.16±0.06
**G**. 3 × (3×3 mg/kg)	0.80±0.42
**H.** 6 × (3×3 mg/kg)	**1.36±0.3**
**Oral administration**	
**I**. 1×0.5 mg/kg	0.05±0.01
**J**. 3 × (3×0.5 mg/kg)	0.09±0.05
**Direct CED into the brain**	
**K.** 2×50 ng	**1.26±0.32**

### Administration of rapamycin

Rapamycin (LC Laboratories, Woburn, MA) was administered intraperitoneally at different doses and regimens to induce the highest achievable levels of GDNF expression (**Table 1**). Rapamycin was first dissolved in N,N-Dimethyl-acetamide (DMAC) and then subsequently diluted for i.p. injection in vehicle consisting of 10% DMAC, 40% polyethylene glycol 400, 40% water, and 10% Tween 80. In addition, two groups of rats (Groups I and J) received Rapamune© (Sirolimus) from Wyeth® Philadelphia, PA by oral gavage (0.5 mg/kg). For direct infusion into the brain (Group K), rapamycin was first dissolved in the above vehicle at a concentration of 4 mg/ml and subsequently serially diluted (1∶100 followed by 1∶16) in PBS to a final rapamycin concentration of 2.5 µg/ml. Twenty microliters (total 50 ng) were infused (CED) into each hemisphere. Conditions for that infusion were identical to CED for the vector delivery. Two daily doses of 50 ng were infused and rats were euthanized 4 days thereafter.

### Individual tissue punch collections

In addition to the above experiments, we created two separate groups of rats in which GDNF level was measured in small (1.5 mm) punches rather than entire striatum. The purpose of this experiment was to demonstrate whether there was any difference between GDNF ELISA performed on the entire striatum results vs restricted tissue punches. Four rats were infused bilaterally (8 hemispheres for analysis) with AAV2-regGDNF (conditions as above). Rats were euthanized after 3 (n = 2) and 6 (n = 2) cycles of rapamycin 3 mg/kg. Punches were taken as described below.

### GDNF enzyme-linked immunosorbent assay (ELISA)

Rats were euthanized and their brains were immediately collected. After dissection, whole striata were stored at –80°C. For the experiment in which individual tissue punches were collected, the freshly removed brains were first snap-frozen in cold (-80°C) isopentane by full immersion. Frozen brains were subsequently cut into 1-mm coronal slices in the rat brain blocker (David Kopf Instruments, Tujunga, CA). Six individual punches were taken from 3 consecutive striatal slices (2 per slice). All punches were taken with an 1.5-mm Harris Uni-Core™, Electron Microscopy Sciences; Hatfield, PA). All samples were later homogenized with a tissue Tearor^TM^, model 985370 (BioSpec Products, Inc., Bartlesville, OK) in 250 µl of ice-cold buffer containing 100 mM potassium phosphate (pH 7.8), 0.2% Triton X-100 and protease inhibitors (Complete Mini®; Roche, Palo Alto, CA) and homogenates were centrifuged at 13,000 x g for 15 minutes. The supernatant solutions were collected and GDNF concentrations measured by ELISA with a commercially available kit (Promega, Madison, WI) as described previously [23].

### Immunohistochemistry

After euthanasia, rats were transcardially perfused with cold PBS and then 4% paraformaldehyde (PA) in PBS. The brains were removed and post-fixed in PA overnight. Brains were washed briefly in PBS, transferred to a 30% w/v sucrose solution in 0.01 M PBS for cryopreservation and then cut into 40-µm serial coronal sections on a cryostat. Frozen sections were collected in a series in antifreeze solution and stored at −80°C. Selected sections were immunostained for GDNF. After 3 washes in PBS, the sections were blocked in 1% v/v H_2_O_2_ for 20 min. After another extensive wash in PBS, sections were incubated in blocking solution (2% v/v normal horse serum and 0.01% v/v Tween-20 in PBS) for 30 min, followed by incubation in primary antibody solution (goat polyclonal anti-GDNF antibody from R&D Systems Inc, Minneapolis, MN; 1∶300 in blocking solution) overnight at room temperature. After three washes in PBS with 0.01% Tween-20, sections were allowed to react for 1 h with biotinylated horse anti-goat IgG (concentration 1∶300; Vector Laboratories, Burlingame, CA), followed by a 1-h incubation in Streptavidin-HRP (1∶300; Vector Laboratories) and a further brief wash in PBS. Immunoreactivity was visualized by DAB/H_2_O_2_ reaction. Sections were mounted onto slides, dried and covered with glass coverslips.

## Results

### Kinetics of GDNF accumulation in the rat brain after transduction with the constitutive AAV2-GDNF vector

**Figure 2:**
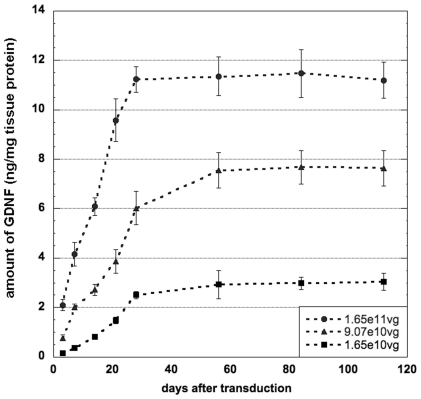
Time-course of GDNF expression in the rat brain after transduction with the constitutive AAV2-GDNF. Three doses of the vector were used and striatal levels of GDNF were measured in the tissue homogenates by GDNF ELISA at various time points after transduction. In all three groups, striatal GDNF increased for the first month and was maintained thereafter at a fixed level for 3 more months. The level of expressed GDNF was proportional to the dose of the vector used.

**Figure 3:**
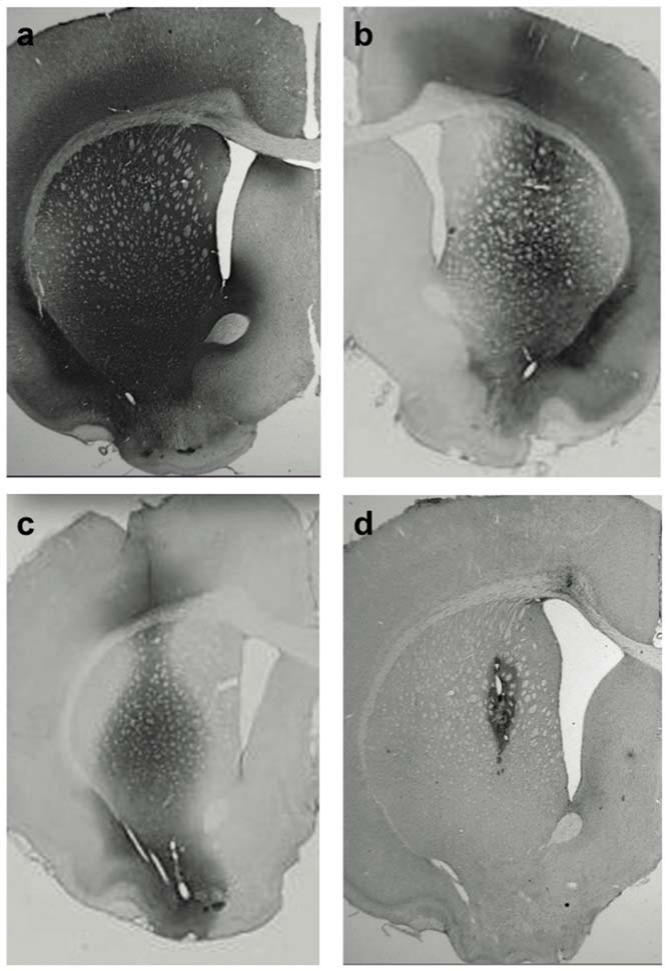
Immunostaining for GDNF expressed in rat striatum from constitutive AAV2-GDNF (a-c) and rapamycin-regulated AAV2-GDNF (d). The constitutive vector was infused at 3 different doses: 16.5×10^10^ vg (**a**), 9.07×10^10^ vg (**b**), and 1.65×10^10^ vg (**c**). The rats were euthanized 4 months after transduction. Robust, widespread signal (proportional to vector dose) confirmed continuous GDNF expression within the injected hemisphere. Much of the signal represents GDNF secreted into the striatal parenchyma. The dose of AAV2-regGDNF was 4.12×10^10^ vg and rats were euthanized 4 days after 3-day Rapamycin regimen (3×10 mg/kg). In contrast to the constitutive vector, the AAV2-regGDNF revealed only focal expression of GDNF localized mainly around the cannula track (**d**). Since this vector induces GDNF secretion only in the presence of rapamycin, the GDNF signal was limited and did not extend beyond the injected striatum (no GDNF accrual upon a single rapamycin cycle).

Initially, GDNF accumulation was analyzed in rat striatum transduced with AAV2-GDNF in which GDNF expression is driven by the constitutive human CMV promoter. Vector was infused into normal rat striata [21], [28] at one of 3 doses (16.5×10^10^, 9.1×10^10^ and 1.65×10^10^ vg). Animals from each group were euthanized at various times and GDNF content measured (**Fig. 1**). In all three groups, striatal GDNF increased for the first month and thereafter was maintained at a fixed level for up to 4 months in all three groups. At the maximum vector dose, mean GDNF content by ELISA was ∼ 11 ng/mg tissue protein. The medium and low doses of vector directed correspondingly lower GDNF tissue concentrations, 6–7 and 2–3 ng/mg tissue protein, respectively (**Fig. 2**). The threshold level of GDNF detectable by the GDNF ELISA kit was ∼ 0.025 ng/mg protein. Immunohistochemical (IHC) evaluation of GDNF expression from the last time-point confirmed robust, widespread, and vector dose-dependent signal within the injected hemisphere (**Fig. 3a-c**).

### Kinetics of GDNF induction from the AAV2-regGDNF induced by rapamycin dosing

**Figure 4:**
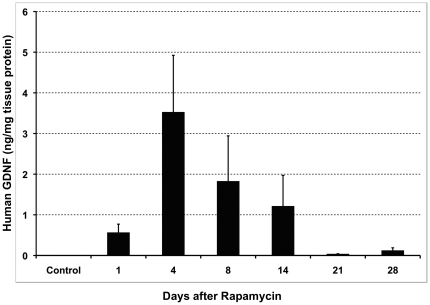
Time-course of GDNF induction from the AAV2-regGDNF induced by rapamycin dosing. Thirty days after transduction, rats received daily intraperitoneal doses of either rapamycin (10 mg/kg) or vehicle (control) for 3 consecutive days. Animals were euthanized and striata processed for GDNF measurements (ELISA) at various days after rapamycin administration. The time-course of GDNF expression suggests that the effect of rapamycin induction on GDNF expression extends out to more than 14 days after the last rapamycin injection, peaking at day 4.

To evaluate the inducible vector system, two vectors, AAV2-FBZhGDNF and AAV2-TF1Nc, were co-infused into rat striatum (see Methods) at a 1∶1 ratio. Thirty days after transduction, rats received daily intraperitoneal doses of either rapamycin (10 mg/kg) or vehicle (control) for 3 consecutive days. Animals were euthanized and striata processed for GDNF content (ELISA) at several time points after rapamycin administration. In the absence of rapamycin, no GDNF could be detected. In contrast, tissues from rapamycin treated animals expressed 3.5±1.4 ng GDNF/mg tissue protein 4 days after the last administration of inducer. GDNF expression detected by IHC revealed only focal expression in the vicinity of the cannula track (**Fig. 3d**). The time-course of GDNF expression suggests that the effect of rapamycin on GDNF expression is persistent, extending out to more than 14 days after the last rapamycin injection. GDNF levels gradually declined from the peak at 4 days to baseline 3-4 weeks later (**Fig. 4**).

### Effect of rapamycin dosing regimen on striatal GDNF concentration

**Figure 5:**
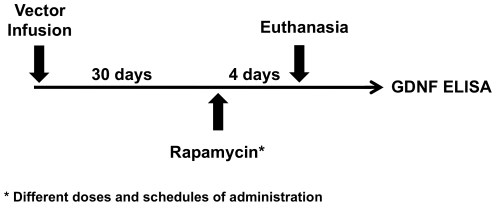
General schedule of rapamycin administration. Thirty days after vector infusion, rats were randomized into 11 groups of 3 rats. Seven groups received intraperitoneal (i.p.) rapamycin; two groups received oral rapamycin and one group received a direct infusion (CED) of rapamycin into the brain. Control animals (n = 3) received i.p. excipient. The detailed rapamycin dosing regimens are listed in **Table 1**.

The time-course of GDNF accumulation after rapamycin administration helped in the design of a general schedule (cycle: 3-days on/4-days off) of rapamycin administration (**Fig. 5**) to test a range of rapamycin dosing regimens. Thirty days after vector infusion, rats were randomized into 11 groups of 3 rats. Seven groups received i.p. rapamycin. Two groups received oral rapamycin (Rapamune®) at 0.5 mg/kg, the maximum dose of rapamycin deliverable orally. One group received a direct parenchymal infusion of rapamycin by convection-enhanced delivery (CED) in two daily doses of 50 ng. Control animals (Group A) received i.p. excipient. The results are summarized in **Table 1**.

### Individual tissue punch collections

The ELISA results from individual punch collections are tabulated in **Table S1** (supplementary data). The mean values are lower than the values generated from the entire striata (**Table 1**).

## Discussion

We evaluated the ability of the dimerizer-regulated gene expression to achieve controlled production of GDNF after striatal administration of AAV2-regGDNF in the rat brain. Our experiments addressed the kinetics of GDNF biogenesis, and accumulated levels with various rapamycin dosing regimens.

All animals that received i.p. rapamycin showed statistically significant increases in striatal GDNF levels (Groups B – H). Oral dosing was less effective (Groups I & J), although 3 cycles of oral drug treatment did produce a significant increase in GDNF (p<0.05) compared to a single cycle. The accumulation of GDNF upon repeat dosing was also evident in i.p. dosing. For example, 2 dosing cycles of rapamycin at 10 mg/kg produced a 5-fold increase in GDNF content (Groups B & C). This schedule of rapamycin dosing produced the greatest tissue concentration of GDNF. However, 10 mg/kg is not a tolerable clinical dose; intravenous doses of rapamycin analogs have only reached 3–5 mg/kg in cancer patients [22], [29], [30]. We therefore investigated chronic dosing with more clinically compatible regimens. Three cycles of 1 or 3 mg/kg (Groups E & G) gave GDNF levels lower than with two cycles of 10 mg/kg. However, 3 mg/kg provided GDNF levels of 0.80 ng/mg protein comparable to levels reported to be neuroprotective against MPTP in marmosets [31], although this study was not designed to evaluate this parameter. This level was further enhanced by 6 cycles of 3×3 mg/kg to 1.4±0.3 ng GDNF/mg protein, approximately 50% of the level obtained after two cycles of 10 mg/kg of rapamycin. These data suggest that high steady-state levels of GDNF in brain tissue can be achieved by chronic rapamycin dosing. Interestingly, direct brain infusion (CED) of rapamycin at a very low dose (50 ng) resulted in GDNF levels of 1.3±0.3 ng GDNF/mg protein, comparable to a 6-week 3×3 mg/kg i.p. rapamycin schedule. This result suggested that rapamycin does not readily cross the blood-brain barrier (BBB). The fact that only a low dose of rapamycin is needed for such induction if delivered directly to the brain suggests perhaps a new strategy for the use of rapamycin-regulated vectors within the CNS in which the vectors are infused into target tissue with an indwelling catheter that delivers very low doses of rapamycin. In addition, new analogues of rapamycin (Deforolimus, AP21967, Ariad Inc.) with higher potency and/or better pharmacokinetic properties could further improve gene regulation and eliminate systemic toxicity. We conclude that a clinically compatible dosing schedule with rapamycin is achievable but it is not clear whether the levels of GDNF obtained are sufficient to promote neuro-regeneration in Parkinsonian primates.

The long-term consequences of high doses of GDNF in PD patients will not be evident until late clinical data become available. Regulation of transgene expression by an FDA-approved drug should provide an additional safety margin and may enable fine tuning of GDNF action at the individual patient level by adjusting the schedule of administered inducer. In our study, repeated induction of GDNF expression from the AAV2-regGDNF clearly resulted in a progressive accumulation of GDNF protein. The experiments were performed with a 3-days on/4-days off regime (cycles) based on the time-course of GDNF expression from AAV2-regGDNF after rapamycin induction. Striatal concentrations of GDNF peaked at day 4 and returned to the threshold of detection 21 days later. This result was very similar to the findings of Auricchio *et al* [32] who used a similar rapamycin-regulated AAV2 system with erythropoietin (Epo) where peak levels were observed at day 3 with return to basal levels by day 21. The highest dose of rapamycin we used (10 mg/kg i.p.) is not acceptable clinically. However, a dose of 3 mg/kg, lower than that used clinically [22], resulted in accumulation of GDNF of 1.4 ng/mg protein (Group H), suggesting that repeat dosing is clinically feasible. It should be noted that 2 ng GDNF/mg protein expressed from the constitutive AAV2-GDNF was the lowest GDNF level that elicited recovery in MPTP-lesioned monkeys [19]. We have not established, however, whether lower steady-state levels of GDNF can also promote recovery in lesioned primates. The present data, nevertheless, demonstrate that the rapamycin-inducible GDNF expression system is fully functional in rats and is able to produce presumptively bioactive levels of GDNF.

It should be noted that whole striata were taken for our analyses (ELISA) to avoid sampling errors from individual punch collections. We define such results as “gross striatal values”. Concerns may be raised regarding the use of entire striata taken for ELISA since, potentially, the readouts might have underestimated the levels of exogenously expressed GDNF (due to averaging and lowering the values of GDNF within the entire striatal tissue). Our data from individual punch collections (**Table S1**) indicated more wide-ranging values (higher standard deviations) with the lower mean than for the data generated from the entire striata (**Table 1**). This confirmed and validated the usage of whole striata for ELISA rather than single tissue snips. “Gross striata values” assure that the entire injected structure is taken into consideration with more precise (total) quantification of GDNF expression.

To further increase the levels of GDNF expressed from the AAV2-regGDNF, higher titers could be used. Our dual component vector system produced lower levels of GDNF than the constitutive vector partly because of the difference in titers. AAV2-regGDNF was infused at a dose of 4.12×10^10^ vg (1∶1 mixture of both vectors at 5.51×10^12^ vg/ml), 4-fold less than the highest dose of the constitutive vector (16.5×10^10^ vg). Immunohistochemical staining of GDNF also confirmed a much wider distribution and robust signal from the constitutive AAV2-GDNF, whereas AAV2-regGDNF showed only focal GDNF expression in the vicinity of the cannula track (Fig. 3). It is important to keep in mind that this rather restricted signal was the result of just one induction cycle (3×10 mg rapamycin/kg) with no GDNF accrual over time (slow tissue clearance [21]). The rats transduced with the constitutive AAV2-GDNF were euthanized after 4 months of continuous production and secretion of GDNF within the rat striatum. Relative strength of promoters is also likely responsible for differences. One of the disadvantages of the two-vector system is also the need to co-transduce neurons with both vectors for regulation to occur. The method of co-transduction with two separate AAV vectors compensates for the restrictions concerning gene insertion into the small AAV vectors. The AAV co-transfection may raise concerns regarding efficiency of transgene expression from two mixed AAV vectors and injected into a single site. Fan *et al.* showed that the tyrosine hydroxylase (TH) gene and also the aromatic L-amino acid decarboxylase (AADC) gene were simultaneously transduced into rat striatal cells via two separate AAV vectors, AAV-TH and AAV-AADC [33]. Immunostaining showed that TH and AADC were co-expressed efficiently in the same striatal cells in vitro and in vivo (>95% double-positive cells were counted). Moreover, co-transduction with these two vectors resulted in more effective dopamine production and more remarkable behavioral recovery in 6-hydroxydopamine (6-OHDA)-lesioned rats, compared with rats receiving AAV-TH alone (p<0.01). Later, the same group tried a triple transduction with AAV's expressing TH, AADC, and GTP Cyclohydrolase I (GCH) [34]. Similarly to their previous results, triple transduction resulted in greater dopamine production in denervated striatum of parkinsonian rats (exceeding double transduction with AAV-TH and AAV-AADC). Molecular studies by Yang *et. al* [35] have demonstrated that intermolecular recombination between monomer circular intermediates (AAV2-GFP and AAV-Alkphos) is, at least in part, responsible for the formation of AAV circular concatamers associated with long-term episomal persistence and transgene expression. New single-vector systems that can accommodate both the regulatory components and the transgene, eliminating the need for co-transduction have already been designed [36], [37], [38]. It should also be emphasized that, for clinical applications, precise targeting and distribution of the vector is warranted. Real-time convective delivery (RCD) of gene therapy vectors has been proposed to address this need [39], [40]. A similar dual-component AAV2-regAADC vector system has also been tested in rat brain [41]. Induction of Aromatic L-Amino Acid Decarboxylase (AADC) expression has been shown in the striatum of unilaterally 6-OHDA-lesioned rats by i.p. injection of rapamycin. Induction of AADC in the lesioned striatum was associated with the development of L-dopa-induced turning behavior. This effect was completely reversible and repeatable. In another long-term (6-year) study of regulated erythropoietin (Epo) expression in NHP skeletal muscle transduced by AAV1-based vectors, Rivera *et al* showed that circulating levels of recombinant Epo are undetectable in the absence of rapamycin or its analogs but could be reliably induced (26 times) to levels that significantly increased hematocrit [37]. Taken together these studies establish that the rapamycin system can be tightly regulated with an appropriate dosing schedule.

If GDNF gene therapy is to become a truly practical mode of treatment of PD, the therapeutic gene will need to be expressed at controlled levels. As we have shown, these levels will depend on the dose of the administered vector and, in case of regulated vectors, the precise dosing regimes of the inducing agents. To our knowledge, we are the first to have made the novel observation that simple changes to rapamycin dosing regimens have dramatic effects on GDNF expression. No research team has yet been able to advance a regulated gene therapy vector into clinical study. Clinical validation of the rapamycin-regulated expression system would be a major innovation in gene therapy, and would likely trigger renewed interest in applications of this approach. As these are only the first steps in controlling expression of transgenes, it is also important to understand the limitations of regulated gene therapy systems before advancing to clinical trials. More studies in animal models should address issues of safety. The ideal solution would be to develop a system that would place a transgene under the control of both a tissue-specific promoter and a disease-specific promoter. The first reports of such advanced systems have already been published [42] and may soon be used in human studies.

## Supporting Information

Table S1 ***In vivo*** **GDNF induction from AAV2-regGDNF after 3- and 6-week of Rapamycin dosing (3 mg/kg) – results from individual striatal punches.** Six individual punches (1.5 mm) were taken from each striatum. The details are described in Materials and Methods.
(PDF)
