# Supplementary material for: Evaluation of an AAV2-Based Rapamycin-Regulated Glial Cell Line-Derived Neurotrophic Factor (GDNF) Expression Vector System
Source: PLoS One. 2011 Nov 21;6(11):e27728. doi: 10.1371/journal.pone.0027728 (PMC3221672; doi:10.1371/journal.pone.0027728)
Supplement: Table S1 — In vivo GDNF induction from AAV2-regGDNF after 3- and 6-week of Rapamycin dosing (3 mg/kg) – results from individual striatal punches. Six individual punches (1.5 mm) were taken from each striatum. The details are described in Materials and Methods. (PDF) [file pone.0027728.s001.pdf]

Table S1. ***In vivo*** **GDNF induction from AAV2-regGDNF after 3- and 6-week of Rapamycin dosing (3 mg/kg) – results from individual striatal punches**

**3 weeks of intraperitoneal administration of Rapamycin (3 mg/kg)**							
Rat / tissue punch	**Striatal GDNF** ng/mg protein	Rat / tissue punch	**Striatal GDNF** ng/mg protein	Rat / tissue punch	**Striatal GDNF** ng/mg protein	Rat / tissue punch	**Striatal GDNF** ng/mg protein
M/L1	**0.85**	M/R1	**0.09**	L/L1	**0.14**	L/R1	0.8
M/L2	**0.24**	M/R2	**0.06**	L/L2	**0.09**	L/R2	0.21
M/L3	**0.33**	M/R3	**0.12**	L/L3	**0.08**	L/R3	0.23
M/L4	**0.11**	M/R4	**0.11**	L/L4	**0.55**	L/R4	0.59
M/L5	**1.49**	M/R5	**1.62**	L/L5	**0.65**	L/R5	0.21
M/L6	**0.15**	M/R6	**0.34**	L/L6	**0.45**	L/R6	0.39
**Mean**	**0.53 ± 0.54**	**Mean**	**0.39 ± 0.61**	**Mean**	**0.32 ± 0.25**	**Mean**	**0.4 ± 0.24**

**6 weeks of intraperitoneal administration of Rapamycin (3 mg/kg)**							
Rat / tissue punch	**Striatal GDNF** ng/mg protein	Rat / tissue punch	**Striatal GDNF** ng/mg protein	Rat / tissue punch	**Striatal GDNF** ng/mg protein	Rat / tissue punch	**Striatal GDNF** ng/mg protein
O/L1	**0.33**	O/R1	**0.35**	P/L1	**0.31**	P/R1	**0.26**
O/L2	**0.86**	O/R2	**0.84**	P/L2	**0.73**	P/R2	**0.27**
O/L3	**0.22**	O/R3	**0.54**	P/L3	**0.42**	P/R3	**0.88**
O/L4	**1.6**	O/R4	**1.43**	P/L4	**0.91**	P/R4	**1.21**
O/L5	**0.66**	O/R5	**1.02**	P/L5	**0.92**	P/R5	**1.81**
O/L6	**1.32**	O/R6	**1.34**	P/L6	**0.43**	P/R6	**1.65**
**Mean**	**0.83 ± 0.54**	**Mean**	**0.92 ± 0.43**	**Mean**	**0.62 ± 0.26**	**Mean**	**1.01 ± 0.66**
